# What can we learn from over a decade of testing bats in New South Wales to exclude infection with Australian bat lyssaviruses?

**DOI:** 10.1111/avj.13143

**Published:** 2022-01-18

**Authors:** TW O'Connor, DS Finlaison, PD Kirkland

**Affiliations:** ^1^ Virology Laboratory Elizabeth Macarthur Agricultural Institute Menangle New South Wales Australia

**Keywords:** Australian bat lyssaviruses, bats, domestic animals, public health, veterinarians

## Abstract

Australian Bat lyssaviruses (ABLV) are known to be endemic in bats in New South Wales (NSW), Australia. These viruses pose a public health risk because they cause a fatal disease in humans that is indistinguishable from classical rabies infection. All potentially infectious contact between bats and humans, or between bats and domestic animals, should be investigated to assess the risk of virus transmission by submitting the bat for testing to exclude ABLV infection. The aim of this study was to establish the prevalence of ABLV infection in bats submitted for testing in NSW and to document any trends or changes in submission and bat details. We examined all submissions of samples for ABLV testing received by the NSW Department of Primary Industries Virology Laboratory for the 13‐year period between 1 May 2008 and 30 April 2021. Fifty‐four (4.9%) ABLV‐infected bats were detected, with some clustering of positive results. This is greater than the prevalence estimated from wild‐caught bats. All bats should be considered a potential source of ABLV. In particular, flying‐foxes with rabies‐like clinical signs, and with known or possible human interaction, pose the highest public health risk because they are more likely to return a positive result for ABLV infection. This review of ABLV cases in NSW will help veterinarians to recognise the clinical presentations of ABLV infection in bats and emphasises the importance of adequate rabies vaccination for veterinarians.

**Abbreviations**
ABLV: Australian bat lyssaviruses
ABLV‐IN: insectivorous variant of ABLV
ABLV‐PT: pteropid variant of ABLV
ACDP: Australian Centre for Disease Preparedness
Ct values: cycle‐threshold
DPI: Department of Primary Industries
EMAI: Elizabeth Macarthur Agricultural Institute
NSW: New South Wales
qRT‐PCR: real‐time reverse‐transcription PCR
RABV: classical rabies virus

Australian bat lyssaviruses (ABLV) are one of 16 species of known lyssaviruses (family *Rhabdoviridae*, genus *Lyssavirus*, species *Australian bat lyssavirus*).^1^, ^2^, ^3^ ABLV have a close antigenic similarity to classical rabies virus (RABV) and commercial vaccines for RABV are protective.^1^, ^4^ Two distinct ABLV variants have been identified in Australian bats, one associated with members of the flying‐fox family^1^ and, another less common variant associated with the insectivorous yellow‐bellied sheath‐tail microbat (*Saccolaimus flaviventris*). These viruses are endemic in New South Wales (NSW) in the respective fruit‐eating or insect‐eating bat populations. Consequently, all bats should be considered a potential source of ABLV.^2^, ^5^, ^6^

Bats infected with ABLV may be more likely to come into contact with humans, or domestic animals. A study in Queensland found a higher prevalence of ABLV in rescued, sick and injured bats.^2^ Some infected bats may appear healthy and behave normally, while other infected bats show clinical signs similar to RABV infection, which include altered vocalisation, sudden and progressive aggression, respiratory difficulties, lordotic spasms or seizure‐like activity and an inability to fly or roost properly.^7^, ^8^ Flying‐foxes experimentally infected with ABLV also displayed signs of muscle weakness, generalised trembling, paralysis of one or more limbs and lethargy.^9^

Domestic animals are susceptible to ABLV. Two cases of natural ABLV infection in horses have been reported in Queensland.^10^, ^11^ Symptoms included progressive hind‐limb ataxia, altered demeanour and mild behavioural changes.^10^ They deteriorated rapidly after the onset of clinical signs and exhibited dysphagia, lethargy and recumbency.^10^ Both horses developed generalised seizures and were subsequently euthanased.^10^ The insectivorous variant of ABLV (ABLV‐IN) was detected and isolated from both the brain and saliva of the infected horses.^11^

Domestic cats and dogs have been experimentally infected with the *Pteropid* variant (ABLV‐PT).^12^ Inoculated cats displayed minimal to occasional behavioural changes such as hissing, hair‐raising and climbing the wire walls of the cage.^12^ Experimentally infected dogs became notably irritated at the site of inoculation and mild hind‐limb ataxia was observed.^12^ All animals had detectable antibodies 2 weeks post infection. However, infection did not advance to the central nervous system of any of the cats or dogs.^12^

Human health is the primary concern when managing incidents of potential ABLV transmission.^5^ Three cases of infection in humans have been confirmed after exposure to bats in Australia.^4^, ^13^, ^14^, ^15^, ^16^, ^17^ Infection is inevitably fatal and clinically indistinguishable from encephalitic rabies.^4^, ^13^, ^14^, ^15^, ^16^, ^17^ Any animal suspected of being infected with ABLV should be tested without delay and, in NSW, these samples should be submitted to the laboratory by a veterinarian.^5^

To date, research into ABLV infection has had an ecological focus to establish the prevalence of ABLV in a range of Australian bat species, or public health strategies to help mitigate the risk of fatal ABLV infection in people.^2^, ^6^, ^16^, ^17^, ^18^, ^19^, ^20^, ^21^, ^22^, ^23^, ^24^, ^25^ In this paper, we review submissions for ABLV testing in NSW over a 13‐year period and investigate the factors associated with bat‐contact, particularly from a veterinary perspective. The objectives were to (1) establish the prevalence of infection in bats submitted over a 13‐year period; (2) provide a clinical description of an ABLV‐infected bat and (3) describe trends in submissions and any potential risk factors for ABLV transmission.

## Materials and methods

Bat carcasses are usually submitted whole to the Elizabeth Macarthur Agricultural Institute (EMAI), for ABLV testing.^5^ All sample submissions are accompanied with a specimen submission form and an electronic copy is stored within the laboratory information management system (SampleManager 12.2.1, Thermo Scientific Waltham, MA, USA).^5^ All submissions for ABLV testing from 1 May 2008 to 30 April 2021 were reviewed. Samples that were not suitable for examination or submissions from a non‐bat species, were excluded from analysis. Reasons for not testing bat samples included animals that were submitted without a head or in a state of such advanced decomposition that an appropriate sample of suitable quality could not be retrieved.

The submission forms were examined to review the clinical presentation of all bats received. The relative frequency for each clinical sign was calculated because more than one clinical sign may have been reported for an individual bat.

To describe trends in submissions received and to identify risk factors associated with ABLV‐infected bats, several details were collated from the submission form. The date when the submission was received was used to categorise the submission by “year” and “season” and the address of the submitting clinic was used to approximate the bat's “location.” Where the information was available, the “species” and “age” of the bat were recorded. Clinical information provided was used to (1) identify the “animal species exposed” to the bat; (2) record if the bat was “reported to be in care or wild” and (3) ascertain a possible “reason for the animal‐bat interaction.”

### Sample collection and testing

On receipt at the laboratory, an ABLV exclusion targeted necropsy was performed by a veterinary pathologist (who has been vaccinated against RABV) and swabs were taken from the oral cavity and freshly cut surface of solid tissues (brain and salivary glands). Both fresh and formalin‐fixed tissues (brain and salivary gland) were also collected. The individual swabs were placed in 3 mL of phosphate‐buffered gelatine saline and tested in separate real‐time, reverse‐transcription PCR assays (qRT‐PCR) to detect both the insectivorous and *Pteropid* variants of ABLV.^26^, ^27^ Details of the nucleic acid extraction and PCR amplification methods and equipment used follow those described elsewhere^28^ except for the substitution of the ABLV specific primers and probes^26^, ^27^ and inclusion of appropriate positive and negative control samples. The baseline was set automatically and the threshold set at 0.05. The cycle‐threshold (Ct) value was used to estimate viral load; a high viral load will result in a low Ct value. Fresh brain and salivary gland were usually referred to the Australian Centre for Disease Preparedness (ACDP), (formerly the Australian Animal Health Laboratory) at Geelong, Victoria for further testing, including detection of ABLV antigens in impression smears using immunofluorescence (IFAT) and testing in the ABLV‐IN or ABLV‐PT qRT‐PCR depending on the species of bat sampled.^5^

### Data analysis

Descriptive and statistical analyses of the data were completed using R (R Core Team^29^). Associations between infection status and the potential risk factors of the submitted bat were investigated using the chi‐squared test (or Fisher's exact test where appropriate). Significance level was set to 0.05, and subsequently, the 95% confidence interval (CI) was calculated for the estimated proportions and odds ratios (OR). The OR was simply calculated using a 2 × 2 table or estimated from univariable logistic regression models.

## Results

Of the 1163 submissions received at EMAI for ABLV testing, 14 bats were excluded as they were unsuitable for testing. A total of 38 submissions were received from non‐bat species, this included testing from 4 feline, 4 bovine, 10 canine, 17 equine and 3 wildlife (possum, koala and deer) submissions. This left a total of 1111 bat submissions received at EMAI for inclusion in the study.

### Prevalence of ABLV infection in bats submitted for testing

From the 1111 bats processed, 54 (4.9%) infected bats were identified. No fresh tissues were available for one ABLV‐infected little red‐flying‐fox (*Pteropus scapulatus*) and diagnosis was made on the detection of antigen by immunohistochemistry at ACDP. For the remaining 53 bats, only the ABLV‐PT variant was detected by qRT‐PCR, with viral RNA detected in all 53 swab samples taken from the brain, 39 (73.6%) swabs taken from the salivary glands and 38 (71.7%) swabs taken from the oral cavity. All cases were confirmed by an ABLV‐PT PCR at ACDP. In contrast, IFAT testing detected antigen in 47/53 (88.7%) fresh brain tissues and 14 (26.4%) salivary gland tissues. Virus isolation was attempted on 49 (92.5%) of the brain tissues referred to the ACDP with infectious virus detected in 45 (84.9%) of the ABLV‐infected bats. Although there were 218 (19.6% of all submissions) insect‐eating bats tested, the ABLV‐IN variant was not detected in NSW during the 13‐year interval reviewed.

**Figure 1:**
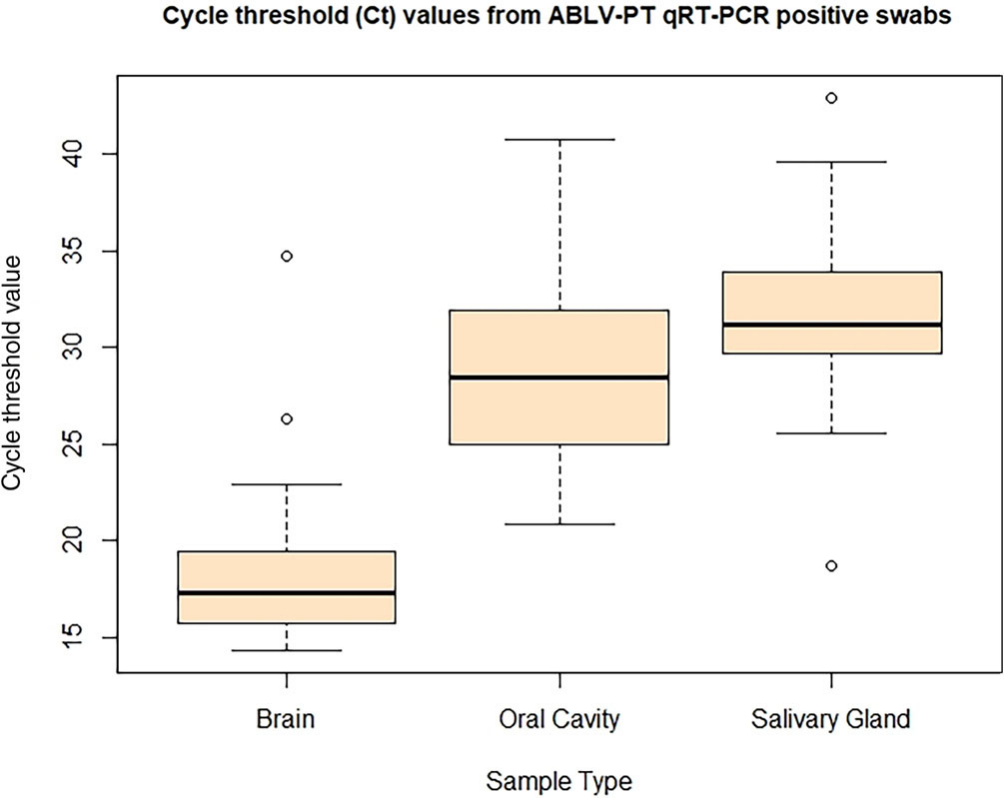
Box and whisker plot depicting the interquartile range and mean cycle‐threshold (Ct) values from positive swabs taken from the brain, oral cavity and salivary glands of ABLV‐infected bats. A lower Ct value indicates a higher concentration of viral RNA. ABLV, Australian Bat lyssaviruses.

The mean cycle‐threshold (Ct) value for swabs taken from brain tissue was 18.7 (range 14.4–34.7); from the oral cavity 28.4 (range 25.2–40.8) and for salivary gland tissue 31.7 (range 18.7–42.9) (Figure 1).

### Clinical presentation of ABLV‐infected bats

A detailed history and clinical signs were available for all but one of the 54 ABLV‐infected bats, however, for three of these bats, no further description of their reported nonspecific, neurological signs was provided. Overall, ABLV‐infected bats were most frequently reported to be found hanging low in a tree, or on the ground, but when compared to bats that gave negative results, infected bats were more frequently reported to have died in care (15.4%), have limited mobility or be unable to use legs–arm to hang or fly (15.4%), be aggressive (10.8%) or emit unusual or altered vocalisations (7.7%). In contrast, bats that have negative results, when compared to ABLV‐infected bats, were more often found dead, or involved in an altercation with a domestic animal (Table 1).

**Table 1:** Clinical signs reported for Australian Bat lyssaviruses (ABLV)‐infected bats listed in the order of frequency (95% confidence interval for the relative frequency provided in parenthesis)

	ABLV‐infected bats		ABLV‐negative bats	
Case history or clinical sign reported	Frequency	Relative frequency of clinical sign (%)	Frequency	Relative frequency of clinical sign (%)
Found low hanging in a tree or on the ground	17	23.3 (22.1–24.5)	95	22.3 (19.8–24.8)
Died in care	11	15.1 (13.9–16.2)	68	16 (13.5–18.5)
**Limited mobility, unable to use legs–arms, unable to hang** ^a^	14	19.2 (18–20.4)	38	8.9 (6.4–11.4)
Incident where a person has been bitten^b^	10	13.7 (12.5–14.9)	178	41.8 (39.3–44.3)
Incident where a person has been scratched^b^	7	9.6 (8.4–10.8)	167	39.2 (36.7–41.7)
**Aggression** ^a^	7	9.6 (8.4–10.8)	28	6.6 (4.1–9.1)
**Emit unusual or altered vocalisations** ^a^	5	6.8 (5.7–8.0)	6	1.4 (0–3.9)
Dysphagia	5	6.8 (5.7–8.0)	20	4.7 (2.2–7.2)
Nonspecific neurological signs	4	5.5 (4.3–6.7)	2	0.5 (0–3)
Found dead^b^	4	5.5 (4.3–6.7)	187	43.9 (41.4–46.4)
Seizures	3	4.1 (2.9–5.3)	18	4.2 (1.7–6.7)
Altercation with a domestic animal^b^	2	2.7 (1.6–3.9)	386	90.6 (88.1–93.1)
Nystagmus	1	1.4 (0–2.5)	2	0.5 (0–3)
Grand Total	73		426	

^a^ Case history or clinical signs, more frequently observed in bats that have tested positive than in bats that test negative.

^b^ Case history or clinical signs, more frequently observed in bats that have tested negative than in bats that test positive.

Signs observed more frequently in infected bats are shown in bold.

Potential infectious human contact, where a person was bitten or scratched by a bat, was recorded for 20 (37.0%) ABLV‐infected bats. However, bats that had not bitten or scratched a person were as likely to be ABLV‐infected as those which had (OR 0.95, CI 0.54–1.67, P‐value 0.96).

### Trends and risk factors observed

#### Year

**Figure 2:**
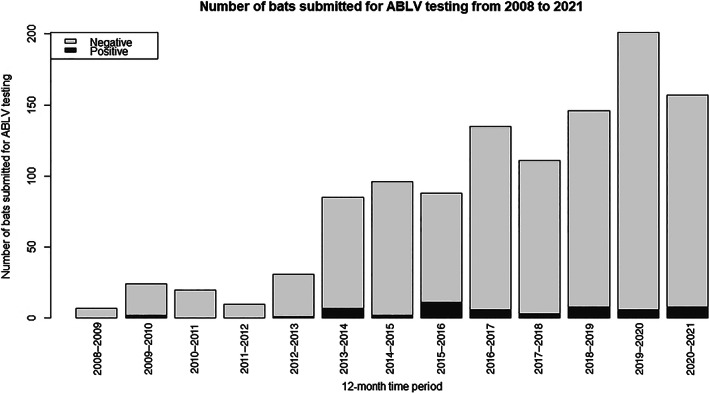
The numbers of bats submitted for ABLV testing from 2008 to 2021. The proportion of positive (ABLV‐infected) bats has remained steady across the 13‐year period (mean 4.7%, median 4.9%, range 3.2 to 5.6%, first quartile 4.3% and third quartile 5.4%). ABLV, Australian Bat lyssaviruses.

Between 2008 and 2021, there has been an increasing number of submissions to EMAI for ABLV testing (Figure 2). A higher proportion of ABLV‐infected bats were detected in 2015–2016 with ABLV‐infected bats making up 12.5% of all submissions received. However, this was not statistically significant (P 0.08). No ABLV‐infected bats were detected in the 12‐month period from May to April 2008–2009, 2010–2011 or 2011–2012.

#### Season

Most bats were submitted in summer (411, 36.9%), followed by autumn (319, 28.7%) and spring (252, 22.7%) with the fewest being submitted in winter (129, 11.6%). There was a similar pattern among the ABLV‐infected bats; however, the mean proportion of ABLV‐infected bats detected in each season remained similar with 23 (5.6%) submitted in summer, 17 (5.3%) in autumn, 8 (3.2%) in spring and 6 (4.7%) in winter. There was no significant association between ABLV diagnosis and the season (P‐value 0.53) in which the bat was submitted.

#### Geographical location

**Figure 3:**
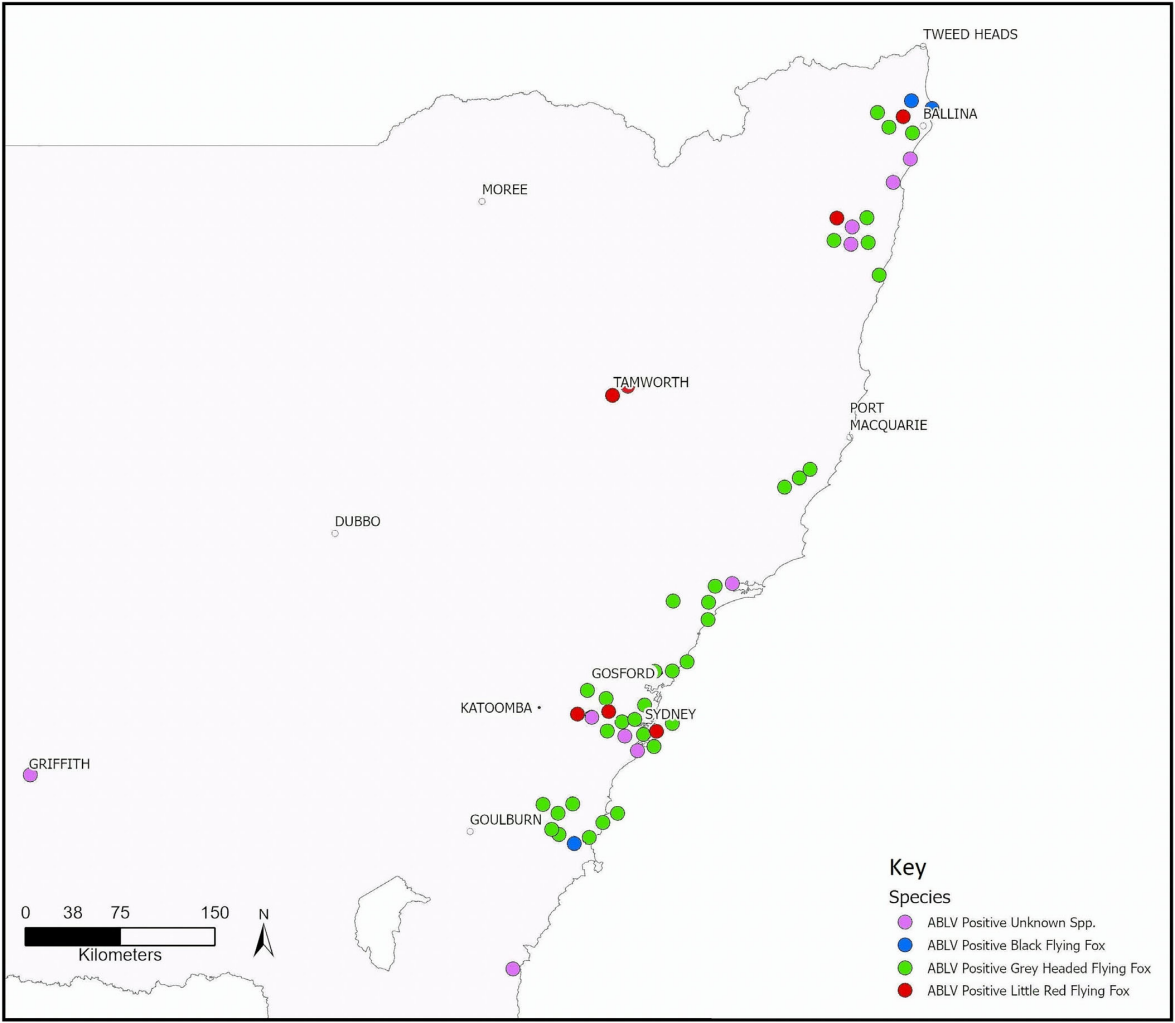
Location and species of ABLV‐infected bats detected on NSW from 2008 to 2021. ABLV, Australian Bat lyssaviruses; NSW, New South Wales.

Submissions were received from 621 different locations, spanning 310 different postcodes. Most submissions were received along the NSW coastline around population centres, with fruit‐eating bats more likely to have been submitted, whereas insect‐eating bats were more likely to have been submitted from inland regions. All but two ABLV‐infected bats were submitted from coastal NSW (Figure 3).

#### Bat species

The suborder of the bat was recorded for 1076 (96.8%) submissions, with 858 identified as fruit‐eating bats and 218 as insect‐eating bats (Table 2). The family of the bat was not identified for one of the 54 ABLV‐infected bats, and the remaining 53 ABLV‐infected bats were flying‐foxes. No ABLV‐infected insect‐eating bats were detected in the study period; while this limits the statistical analysis for risk factors associated with bat species to those within the flying‐fox suborder, it is obvious that flying‐foxes submitted for ABLV testing are more likely to be infected.

**Table 2:** Species of bats submitted for Australian Bat lyssaviruses (ABLV) testing and number, proportion and 95% confidence intervals (CI) of ABLV‐infected bats for each species included in parentheses

Species	Number of bats identified (number infected)
Fruit‐eating bats – total 858 of which the species were identified as:	
Grey‐headed flying‐fox (*Pteropus poliocephalus*)	463 (34; 3.4%, CI 0%–7.1%)
Black flying‐fox (*Pteropus alecto*)	89 (3; 7.3%, CI 4.9%–9.7%)
Little red flying‐fox (*Pteropus scapulatus*)	42 (7; 16.7%, CI 5.4%–27.9%)
Common blossom bat (*Syconycteris australis*)	2
Spectacled flying‐fox (*Pteropus conspicillatus*)	1
Species not identified	259 (9; 3.5%, CI 1.2%–5.7%)
Insect‐eating bats – total 218 of which the following species were identified:	
Lesser long‐eared bat (*Nyctophilus geoffroyi*)	13
Ghost bat (*Macroderma gigas*)^a^	5
Gould's wattled bat (*Chalinolobus gouldii*)	4
Little forest bat (*Vespadelus vulturnus*)	3
Gould's long eared microbat (*Nyctophilus gouldi*)	2
Eastern horseshoe bat (*Rhinolophus megaphyllus*)	1
Eastern free‐tail bat (*Molossidae* spp.)	1
Inland free‐tailed bat (*Mormopterus petersi*)	1
Southern Myotis (*Myotis macropus*)	1
Yellow‐bellied Sheathtail‐bat (*Saccolaimus flaviventris*)	1
Species not identified	186
Suborder not identified for 35 bats (including one ABLV‐infected bat)	

^a^ Bats kept as part of a zoological collection in NSW.

Of the 858 flying‐foxes submitted, 34 (3.4%) of the 463 grey‐headed flying‐foxes (*Pteropus poliocephalus*), 7 (16.7%) of the 42 little red flying‐foxes and 3 (7.3%) of the 89 black flying‐foxes (*Pteropus alecto*) were ABLV‐infected (Table 2). When comparing the number of bats recorded for each species, although the number of cases was small, the proportion of ABLV‐infected little red flying‐foxes (CI 5.4–27.9%, P‐value 0.04) was higher than for other species.

#### Age

The estimated age was available for 361 (32.5%) of the bats submitted for testing; 235 were identified as adult, 117 were juveniles and 9 were neonates. Among the ABLV‐infected bats, 19 were recorded as adults and 9 were young or juvenile. No estimate of age was provided for 28 infected bats. No statistical association (OR 1.0, CI 0.5–2.4, P‐value 1.0) was found when comparing the ABLV infection status of bats in the adult and juvenile groups.

#### Species exposed to bat

**Figure 4:**
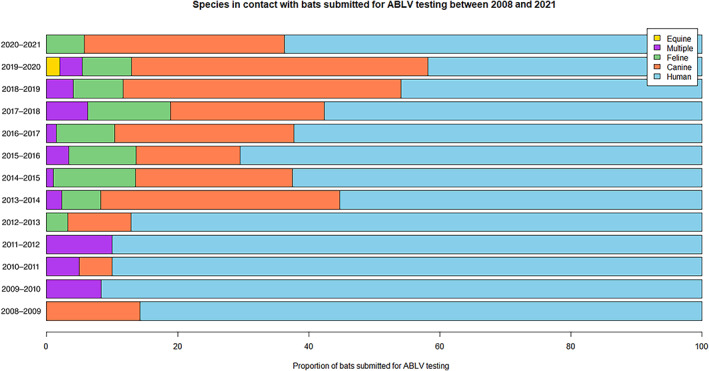
In‐contact species for ABLV submissions received in each 12‐month period from 2008 to 2021. While most bats submitted for ABLV testing are due to human contact (58.5%), this proportion has steadily decreased over time. ABLV, Australian Bat lyssaviruses

Most submissions arose from human contact (650, 58.5%) followed by canine (337, 30.3%) and then feline contact (88, 7.9%). Thirty‐two (2.9%) bats had contact with multiple species. Potential exposure in horses was also investigated in 4 (0.4%) submissions. The proportion of ABLV testing from human contact has declined from 2013, whereas the proportion of submissions from bat‐contact with a domestic animal has increased (Figure 4).

Among the ABLV‐infected bats, human exposure was reported for 51 (94.4%) bats and canine contact was noted for 3 (5.6%) ABLV‐infected bats. Bats submitted for ABLV testing after human exposure were more likely to be positive when compared to bats with canine contact (OR 9.5, CI 2.9–30.6, P‐value <0.001).

#### In care or wild at the time of exclusion

Most (944, 84.9%) of the bats submitted for ABLV testing were reported to be wild caught and 167 (15.0%) bats were in care or captivity at the time of submission. In contrast, 31 (57.4%) ABLV‐infected bats were reported to have been in care at the time of submission. Bats in care at the time of submission were statistically more likely to be ABLV‐infected compared to bats that were reported to be wild (OR 9.1, CI 5.2–16.1, P‐value <0.001).

#### Reason for bat interaction

Where histories were provided, the majority of bats were submitted due to perceived ABLV risks to a pet. This was split into two situations: when a pet attacked a bat (232, 20.9%) and when a pet was found with a bat (224, 20.1%). There were 139 (12.5%) bats submitted due to human contact that could lead to infection after attempted rescue from entanglement (in barbed wire, fruit netting or fences). Other reasons for submission included 102 (9.2%) bats that had exhibited unusual behaviours or clinical signs, 67 (6.0%) bats that were found dead, 50 (4.5%) bats that had died while in care and 38 (3.4%) bats that had undergone some form of veterinary treatment (including radiographs, fluid therapy and euthanasia). There were 192 (17.3%) bats where no description of an interaction was provided.

Among the ABLV‐infected bats, 23 (42.6%) had exhibited unusual behaviour or clinical signs, 10 (18.5%) died while in care, 4 (7.4%) bats were associated with mass mortality events, 2 (3.7%) were submitted due to a pet attack, 2 (3.7%) bats underwent some form of veterinary treatment and 1 (1.9%) bat was submitted due to potentially infectious human contact after attempted rescue from entanglement. There were 12 (22.2%) ABLV‐infected bats where no description of an interaction was provided. Bats submitted for testing with unusual behaviour (estimated OR 9.0, CI 3.6–23.1, P‐value <0.001) or died while in care (estimated OR 7.8, CI 2.7–22.7, P‐value <0.001) were more likely to be ABLV‐infected when compared to bats with no description of interaction provided.

## Discussion

In our analysis of submissions for ABLV testing received in NSW from 2008 to 2021, we have established the prevalence of infection in bats submitted over a 13‐year period, provided clinical descriptions of ABLV‐infected bats and identified risk factors associated with ABLV infection of bats.

The number of ABLV submissions received at EMAI has steadily increased over the 13‐year period covered by this study (Figure 2). This corresponds with the increased number of notifications made to NSW public health officials and an increased number of residents seeking postexposure treatment in summer.^20^, ^21^, ^30^ Nationally, summer and autumn are reported to be the highest risk period for ABLV infection. In NSW, although statistically the risk of ABLV transmission remains the same regardless of the season of submission, the general trend is consistent with the national situation, that is, there is also a higher frequency of bat‐human interactions in summer and autumn.^25^

Our data confirm that there are risks to both human and animal health, with infected bats found over a wide geographical area along the NSW coast where there are many large population centres, including cases in the suburbs of Sydney (Figure 3). ABLV‐infected grey‐headed flying‐foxes were found throughout their range in NSW, including in parks of central Sydney, indicating the potential risk to humans and especially children.^19^ The detection of an ABLV‐infected black flying‐fox, at Bomaderry, on the NSW South Coast, demonstrates the extensive range of flying‐foxes in NSW. There is an increasing risk of infections in southern coastal regions of NSW and Victoria as the range of flying‐foxes expands under the influence of rising temperatures.^31^, ^32^

Our study confirms that bats submitted for ABLV testing are more likely to be infected than those tested during structured surveys of wild‐caught bats.^2^, ^25^ The prevalence of ABLV in wild populations is estimated to be less than 1%, whereas we have found a prevalence of 4.9% in bats submitted for ABLV testing. This is consistent with the prevalence of infection reported for bats submitted in Queensland (6.8%) and also nationally (4.5%).^2^, ^19^, ^23^ The failure to detect ABLV in insect‐eating bats in this review is consistent with observations in Queensland^2^, ^6^, ^18^, ^23^ and may be related to a combination of their more elusive behaviour and that they occupy an ecological habitat that results in a lower likelihood of contact with humans and hence submission for testing.^1^, ^2^ However, insect‐eating bats are still a potential source of ABLV and have been implicated as the source of infection for horses in Queensland.^2^, ^6^, ^18^, ^23^

When the species of bat was available, most ABLV‐infected bats were identified as grey‐headed flying‐foxes. This is not surprising because the grey‐headed flying‐fox is the most abundant species in NSW. However, nationally, the little red flying‐fox is considered to have a higher prevalence of infection than other flying‐fox species.^2^, ^25^ While statistically a higher proportion of little red flying‐foxes were ABLV‐infected (P‐value 0.04) in our study, the estimate was based on a small sample size suggesting that human contact with this species was less frequent in NSW. Therefore, from an overall risk perspective, the little red flying‐fox should not be considered differently to any other species and all bats in NSW should be considered a potential source of ABLV.

ABLV‐infected bats were more frequently in care at the time of investigation. They were often found in unusual circumstances (e.g., limited mobility, unable to use legs–arms, unable to hang) and exhibiting a range of neurological signs that could elicit human intervention and care. This is consistent with national bat surveillance data.^25^

This is the first report of the differences in viral load detected in samples of brain, oral cavity and salivary gland in ABLV‐infected bats (Figure 1). The highest virus loads were consistently detected in brain samples, similar to results for wild bats infected with European bat lyssavirus 1.^33^ European bat lyssavirus 1 was only detected in the brains of those animals with neurological disease and, of these infected bats, all but one showed oropharyngeal excretion.^33^ This high viral load in brain samples emphasises the need for this tissue to be sampled for ABLV testing. The detection of moderate levels of ABLV in both the salivary glands and swabs of the oral cavity emphasises the high risk of transmission if an animal or person is bitten or scratched by a bat.

The current “gold standard” for laboratory confirmation of ABLV infection is the detection of viral antigen in brain tissues by IFAT.^34^ However, antigen detection assays are not quantitative, are not suited to testing of swab samples and lack the analytical sensitivity and sometimes the specificity of qRT‐PCR. This is highlighted by the IFAT returning a false negative result for six (11.3%) ABLV‐infected brains that tested qRT‐PCR positive. However, depending on the selection of test reagents, IFAT can provide a capacity to screen samples for novel variants of ABLV.

Despite aggression being reported more often for ABLV‐infected bats, uninfected bats were reported to bite just as frequently. Most bat‐related injuries occur when people attempt to rescue a bat or to try to release a bat from entanglement.^30^ Given that moderate quantities of ABLV have been detected in the salivary glands and oral cavity, and infectious virus has been isolated from 84.9% of ABLV‐infected bat brains, any transmucosal or transdermal contact (if there is a break in the skin barrier) with saliva from an infected bat could result in virus transmission.

Individuals caring for bats, and those submitting bats for investigation, are at a higher risk of being exposed to ABLV and should be adequately vaccinated.^2^, ^19^ For veterinary practice, the importance of adequate rabies (RABV) vaccination cannot be overstated. Our study indicates that veterinarians and associated clinic staff are among the people most likely to encounter an ABLV‐infected bat both because of the disease investigation process and the potential for high‐risk contact with bats (such as bites, scratches and needle‐stick injuries) that can occur during treatment and euthanasia. A survey in Queensland in 2018 established that only 31.5% of veterinarians had been vaccinated in the past and only 15.5% considered themselves currently vaccinated.^24^ Given the greater risk of exposure and the increasing number of submissions, veterinary practices should review the vaccination status of all staff.

Bat‐contact with a pet is of public health interest because surveys in adults reported that 50% of participants would handle a bat if a pet was interacting with it.^16^, ^22^ In our study, a growing proportion of bats were submitted because of bat‐contact with a domestic animal and 41% of submissions were received due to concern for pet safety. Nationally, pet contact was also the most common reason for testing, comprising 33.7% of submissions.^25^ In the last 5 years, there has been an increase in the proportion of submissions received due to a domestic animal exposure to a bat (Figure 4). Some of this increase may be the result of two cases of confirmed ABLV infection in horses in 2013 and the infection of an 8‐year‐old boy in the same year, both of which are likely to have generated greater awareness of the risks of ABLV posed by bats to humans and domestic animal species.^11^, ^13^ In addition, domestic animals may be encountering bats more often.^35^, ^36^, ^37^ Rabies vaccination for dogs and cats, after possible contact with an infected bat, no longer requires approval from the NSW Chief Veterinary Officer.^5^ Appropriate rabies vaccination of a pet after bat‐contact should help mitigate the risk of ABLV transmission from domestic animals to humans.^5^

## Conclusion

There is a substantially higher prevalence of ABLV in bats submitted for ABLV testing than has been reported from surveys of wild‐caught animals. Bats with potentially infectious human contact, where a person was bitten or scratched by a bat, especially those with clinical signs suggestive of ABLV infection, pose a public health risk and should be submitted for exclusion of ABLV infection. Veterinarians and individuals caring for bats are at a higher risk of being exposed to ABLV and should be adequately vaccinated. We found that a growing proportion of bats were tested as a result of contact with a domestic animal. Recent changes to improve the availability of rabies vaccination for dogs and cats after potentially infectious contact with a bat should help mitigate the risk of infection of domestic animals. While the risk of secondary transmission of ABLV to humans from pets is low, the risk of human infection directly from infected bats cannot be ignored.

## Conflicts of interest and sources of funding

The authors declare no conflicts of interest or sources of funding for the work presented here.
